# Daily Fluctuation of Orexin Neuron Activity and Wiring: The Challenge of “Chronoconnectivity”

**DOI:** 10.3389/fphar.2018.01061

**Published:** 2018-09-25

**Authors:** Idris A. Azeez, Federico Del Gallo, Luigia Cristino, Marina Bentivoglio

**Affiliations:** ^1^Department of Neuroscience, Biomedicine and Movement Sciences, University of Verona, Verona, Italy; ^2^CNR-Institute of Biomolecular Chemistry, Pozzuoli, Italy; ^3^National Institute of Neuroscience, Verona Unit, Verona, Italy

**Keywords:** hypocretin, melanin-concentrating hormone, sleep, wake, synaptic plasticity

## Abstract

In the heterogeneous hub represented by the lateral hypothalamus, neurons containing the orexin/hypocretin peptides play a key role in vigilance state transitions and wakefulness stability, energy homeostasis, and other functions relevant for motivated behaviors. Orexin neurons, which project widely to the neuraxis, are innervated by multiple extra- and intra-hypothalamic sources. A key property of the adaptive capacity of orexin neurons is represented by daily variations of activity, which is highest in the period of the animal’s activity and wakefulness. These sets of data are here reviewed. They concern the discharge profile during the sleep/wake cycle, spontaneous Fos induction, peptide synthesis and release reflected by immunostaining intensity and peptide levels in the cerebrospinal fluid as well as postsynaptic effects. At the synaptic level, adaptive capacity of orexin neurons subserved by remodeling of excitatory and inhibitory inputs has been shown in response to changes in the nutritional status and prolonged wakefulness. The present review wishes to highlight that synaptic plasticity in the wiring of orexin neurons also occurs in unperturbed conditions and could account for diurnal variations of orexin neuron activity. Data in zebrafish larvae have shown rhythmic changes in the density of inhibitory innervation of orexin dendrites in relation to vigilance states. Recent findings in mice have indicated a diurnal reorganization of the excitatory/inhibitory balance in the perisomatic innervation of orexin neurons. Taken together these sets of data point to “chronoconnectivity,” i.e., a synaptic rearrangement of inputs to orexin neurons over the course of the day in relation to sleep and wake states. This opens questions on the underlying circadian and homeostatic regulation and on the involved players at synaptic level, which could implicate dual transmitters, cytoskeletal rearrangements, hormonal regulation, as well as surrounding glial cells and extracellular matrix. Furthermore, the question arises of a “chronoconnectivity” in the wiring of other neuronal cell groups of the sleep-wake-regulatory network, many of which are characterized by variations of their firing rate during vigilance states.

## Introduction

A wealth of studies has been stimulated by the discovery, two decades ago, of neurons of the lateral hypothalamus (LH) which synthesize the orexin/hypocretin peptides (de Lecea et al., 1998; Sakurai et al., 1998). Interest in these neurons has been boosted by findings that rapidly accumulated on their role in food intake, in the promotion and consolidation of wakefulness, as well as in a wide range of other physiological functions relevant for motivated behaviors.

These functions require capacity of adaptation to the internal milieu of the organism and to the external environment. Daily variations of activity, demonstrated for orexin neurons, are essential for such adaptation. At the synaptic level, adaptation should be subserved by plasticity. Seminal investigations have shown plastic synaptic changes in the excitatory wiring of orexin neurons after changes in food intake or prolonged wakefulness (Horvath and Gao, 2005; Rao et al., 2007). Such findings have focused attention on the response of these neurons to perturbations of physiological functions. The present review wishes to highlight that plastic changes of the wiring of orexin neurons occur also in unperturbed animals over the course of the day and in relation with sleep or wakefulness.

The orexin system and sources of inputs to orexin neurons are here first summarized. This is followed by a review of different lines of evidence pointing to daily variation of the activity of orexin neurons and to remodeling of their synaptic wiring in response to challenges. Dynamic changes in the synaptology of orexin neurons in basal conditions are then discussed. Altogether the findings point to daily variations in the excitatory/inhibitory wiring of orexin neurons as operational mode of these neurons inserted in a network that should meet daily demands.

## Overview of the Orexin System

The orexin system has been the subject of authoritative reviews (e.g., Yamanaka et al., 2003a; Sakurai, 2007, 2014; Sakurai and Mieda, 2011; Kukkonen, 2013; Bonnavion et al., 2016; Peyron and Kilduff, 2017; Tyree et al., 2018) and only a brief synopsis is here presented.

Orexin-A and orexin-B are also named hypocretin-1 and -2, and the double nomenclature is due to the designation of the molecules at the time of their discovery. These paired peptides are synthesized by neurons located in the perifornical area and LH (Peyron et al., 1998). The orexins are cleaved by a proteolytic process from the common precursor prepro-orexin, a 130 amino acid molecule encoded by a single gene (located in humans in chromosome 17), into two smaller peptides, which share 13 amino acid identities and are highly conserved across species in mammals and non-mammalian vertebrates (Kukkonen, 2013).

Orexin-A and B are largely colocalized in the same neurons, as shown in the human (Thannickal et al., 2000) and rodent (Nixon and Smale, 2007) brain. Orexin-A is more stable and lipophilic than orexin-B (Kastin and Akerstrom, 1999), and can be measured in the cerebrospinal fluid (CSF). We will here refer to orexin-A or both orexin-A and -B as orexin, unless explicitly specified.

Orexin neurons represent a relatively small population: about 7,000 neurons in the rat brain (Modirrousta et al., 2005) and 50,000–80,000 neurons in the human brain (Thannickal et al., 2000; Steininger and Kilduff, 2005).

Orexin actions are mediated by two membrane-bound G protein-coupled receptors: orexin-1 receptor (OX1R) and orexin-2 receptor (OX2R) (Sakurai et al., 1998; Inutsuka and Yamanaka, 2013). OX1R is linked to excitatory G proteins of the Gq subclass, and OX2R can signal through Gq or inhibitory Gi/Go proteins. OX1R has greater affinity for orexin-A over orexin-B, whereas OX2R accepts both ligands with similar affinities. The different complementary distribution of *OX1R* and *OX2R* mRNAs suggests that these receptors enhance distinct physiological roles in diverse brain regions (Marcus et al., 2001).

The study of the distribution of orexin fibers rapidly revealed that they establish a rich network within the LH and other hypothalamic regions, including the dorsomedial and ventromedial hypothalamic nuclei, arcuate nucleus, as well as the wake-promoting histaminergic tuberomammillary nucleus (TMN) (Peyron et al., 1998; Eriksson et al., 2001; Peyron and Kilduff, 2017). Orexin projections extend widely beyond the hypothalamus, innervating the neocortex and hippocampus, forebrain structures implicated in the processing of emotion and motivation, such as the amygdala, nucleus accumbens, bed nucleus of the stria terminalis (BNST), and ventral tegmental area (Peyron et al., 1998). In the thalamus, orexin axons course along the midline and are densely distributed in the thalamic paraventricular nucleus (review in Colavito et al., 2015). Targets of orexin fibers in the brain stem include key nodes in sleep-wake regulation: noradrenergic neurons of the locus coeruleus and serotonergic neurons of the dorsal raphe (Peyron et al., 1998; Horvath et al., 1999). Descending orexin fibers are distributed to all segments of the spinal cord, densely innervating the intermediolateral cell column, with a moderately dense distribution to the ventral horn (van den Pol, 1999). Orexin fibers have been found to be apposed to motoneuron cell bodies, as described at lumbar spinal levels (Yamuy et al., 2004).

The orexin peptides were first recognized as regulators of feeding behavior (Sakurai et al., 1998) and orexin neurons are connected to hypothalamic regions that regulate energy balance. The targets mentioned above indicated that these neurons are also inserted in the network of sleep-wake regulation. Demonstrating a role of orexin in this function, features of the sleep disorder narcolepsy were found in murine models of deficient orexin signaling (Chemelli et al., 1999; Lin et al., 1999). Interest in the involvement of orexin neurons in sleep-wake regulation was also stimulated by the finding that they degenerate in the brain of subjects affected by narcolepsy (Thannickal et al., 2000) and in particular the form of narcolepsy with cataplexy currently classified as narcolepsy type I (Scammell, 2015). Optogenetic investigation has demonstrated a role of orexin in the regulation of sleep-wake transitions (Adamantidis et al., 2007).

Of note, orexin neurons link limbic regions and arousal-regulating regions, and the activation of orexin neurons by the limbic system could be instrumental in maintaining wakefulness during emotional arousal (Sakurai, 2014). In humans, increased levels of orexin, measured in the amygdala, have been found during social interactions and in connection with social-induced positive emotions (Blouin et al., 2013).

The orexin system also regulates autonomic functions and the neuroendocrine axes, as well as plasticity mechanisms related to reward (López et al., 2010; Giardino and de Lecea, 2014; Baimel et al., 2015). Furthermore, the orexin system has been implicated in cognitive processes, e.g., in the facilitation of hippocampal-dependent learning and memory functions (Aitta-Aho et al., 2016; Mavanji et al., 2017), and in the modulation of γ-aminobutyric acid (GABA) and glutamate release in the hippocampus (Stanley and Fadel, 2012), in fear memories (Flores et al., 2014) and in the modulation of amygdala-dependent aversive memory formation (Sears et al., 2013).

Furthermore, orexin neurons innervate brain regions related to nociception, especially the periaqueductal gray (Hagan et al., 1999; Ho et al., 2011), and play a role in the link between nociception and analgesia (Cristino et al., 2016; Inutsuka et al., 2016; Razavi and Hosseinzadeh, 2017).

These broad range of functions has led to the definition of orexin neurons as “physiological integrators” (de Lecea et al., 1998; Inutsuka and Yamanaka, 2013) and “multitasking” (Sakurai, 2014).

### Multitasking Neurotransmission

Orexin neurons are multitasking also concerning neurotransmitters. Orexin is excitatory (de Lecea et al., 1998) and a wealth of evidence indicates that orexin neurons co-release the excitatory neurotransmitter glutamate. It was initially reported that orexin cell bodies show glutamate immunoreactivity (Abrahamson et al., 2001), and optogenetic activation has more recently shown glutamate release (Schöne et al., 2014). A recent study in genetically engineered mice has reported that about 86% of orexin neurons express the vesicular glutamate transporter-2 (VGluT2), a marker for glutamate-releasing neurons (Blanco-Centurion et al., 2018).

In early ultrastructural studies, orexin axon terminals in the TMN were found to establish asymmetric synapses (Torrealba et al., 2003), though both symmetric and asymmetric contacts of orexin boutons were also reported (Yamanaka et al., 2002). Asymmetric synapses and some symmetric synapses established by orexin fibers have been observed in other targets (review in Ma et al., 2018). It was also shown that at the presynaptic terminal glutamate is stored in small, clear, synaptic vesicles, whereas orexin is contained in large, dense core vesicles, which suggested a different regulation of the release of the fast neurotransmitter and the peptide (Torrealba et al., 2003). Fast glutamatergic control of orexin axons in the TMN (Schöne et al., 2012), with a differential regulation of glutamate and orexin spike outputs was then actually shown by optogenetic stimulation (Schöne et al., 2014), and this confirmed glutamate and orexin co-transmission in targets of orexin efferents.

There are also findings indicating that a subset of orexin neurons (about 10–20%) may release GABA, and that they exert a direct local inhibitory effect on neurons which contain melanin-concentrating hormone (MCH; see further) (Apergis-Schoute et al., 2015). By single-cell gene profiling expression of the gene encoding glutamate decarboxylase (GAD, the GABA synthetic enzyme) 65 was found in about 50% of orexin neurons, which would therefore be potentially capable of GABA synthesis (Mickelsen et al., 2017). This adds complexity to potential inhibitory effects of orexin neurons, which could also be mediated by disinhibition of local GABAergic neurons in the LH (e.g., Belle et al., 2014; Apergis-Schoute et al., 2015).

Orexin neurons also co-express other peptides (review in Ma et al., 2018). Among these, the colocalization with the inhibitory peptide dynorphin (Chou et al., 2001) seems to be of particular interest for synergistic functional effects of local release (Ferrari et al., 2018), as outlined below.

### Wiring of Orexin Neurons: Extra- and Intra-hypothalamic Inputs

The normal synaptic organization of orexin neurons has been initially examined in intact mice used as controls of manipulations of food intake (Horvath and Gao, 2005). This study was based on electrophysiological approaches (whole-cell recording in lateral hypothalamic slices from mice in which orexin neurons are tagged by green fluorescent protein, GFP), ultrastructure (identification of asymmetric, i.e., putative excitatory, and symmetric, i.e., putative inhibitory, synapses), fluorescence immunostaining of presynaptic components using VGluT2 to label excitatory axon terminals and GAD to label GABAergic ones. Altogether these approaches provided evidence in control animals (fed *ad libitum*) of a dense excitatory innervation of orexin cell bodies: immunofluorescence showed a 5:1 ratio of excitatory/inhibitory perisomatic input, and electron microscopy showed a ratio of about 2:1 of asymmetric versus symmetric contacts on dendrites. Excitatory synaptic currents were consistent with the prevalence of excitatory innervation of orexin neurons. Furthermore, dendro-dendritic interactions were prominent among orexin-immunopositive shafts and unidentified dendritic shafts of LH neurons. Since the receptor for the adipose hormone leptin is expressed at orexin neurons, a relationship between the levels of circulating leptin and the synaptic arrangement was hypothesized and verified with paradigms of fasting and re-feeding and leptin administration. The animals were maintained under a 12 h/12 h light/dark (LD) cycle. Although the time of sacrifice was not explicitly mentioned, on the basis of the experimental protocol (overnight food deprivation) the animals were presumably sacrificed at the end of the period of darkness.

The predominantly excitatory synaptic organization of orexin neurons described in this study was defined as “unorthodox” compared to other neuronal cell types in the central nervous system (Horvath and Gao, 2005), especially concerning perisomatic innervation. For example, in pyramidal cells of the CA1 hippocampal field and neocortex, the cell body receives GABAergic inhibitory terminals, whereas most of the excitatory inputs target dendrites (Megías et al., 2001; Spruston, 2008).

The sources of excitatory and inhibitory afferents to orexin neurons are diverse and represent a complex chapter of their connectivity and functional effects. Afferents have been investigated by methods with increasing selectivity and sensitivity: conventional tract tracing techniques, genetic tracing, neurochemical identification of boutons with multiple immunofluorescence, optogenetics. Altogether this wealth of data has shown that neural inputs originate from a variety of extra-hypothalamic sources, which do not always reciprocate orexin projections, and from intra-hypothalamic sources, including a dense network within the LH.

In particular, overall mapping of afferents to orexin neurons has been performed with anterograde tract tracing based on injections of biotinylated dextran, combined with retrograde tracing with cholera toxin B subunit in rats (Yoshida et al., 2006), and retrograde tracing using transgenic mouse lines which express tetanus toxin C fragment fused to GFP (*TTC::GFP* transgene) in orexin neurons (Sakurai et al., 2005). These studies have shown that cortical input derives from the limbic (infralimbic and prelimbic) cortex, and dense afferents originate from the basal forebrain, amygdala, septum, nucleus accumbens and BNST. Many hypothalamic sources have been identified: preoptic area, GABAergic neurons of the sleep-promoting ventrolateral preoptic nucleus (VPLO), posterior hypothalamus, arcuate nucleus and LH. Afferents from the brain stem derive mainly from cholinergic neurons of the mesopontine nuclei and serotonergic neurons of the raphe nuclei.

Concerning the abundant input from the basal forebrain (Henny and Jones, 2006), conditional anterograde tracing of neurotransmitter-identified circuits has shown that the substantia innominata sends glutamatergic and GABAergic fibers to orexin neurons (Agostinelli et al., 2017). Optogenetic stimulation has shown that this glutamatergic input excites orexin neurons, whereas, surprisingly, the GABAergic input, though equally dense, rarely forms functional synapses (Agostinelli et al., 2017).

Glutamatergic axons derived from local neurons and/or other hypothalamic and extra-hypothalamic sources have been reported to account for excitatory inputs to orexin neurons (Li et al., 2002). Electrophysiological recordings from slice preparations showed that orexin neurons are activated by agonists of ionotropic glutamate receptors and inhibited by glutamate antagonists (Li et al., 2002; Yamanaka et al., 2003b), indicating that orexin neurons are tonically activated by glutamate (review in Inutsuka and Yamanaka, 2013).

The local network in the LH, which is formed by heterogeneous neuronal subsets (Gerashchenko and Shiromani, 2004; Burt et al., 2011) plays an important role in orexin neuron activity regulation. Results from immuno-electron microscopy analyses and patch-clamp recordings in slices have shown that orexin neurons directly innervate each other, and are indirectly activated via OX2R (Yamanaka et al., 2010). Furthermore, excitatory inputs to orexin somata originate from hypothalamic neurons which produce corticotropin releasing hormone, and this could represent a pathway for stress-induced activation of the orexin system, given the role of these neurons in the response to stress (Winsky-Sommerer, 2004). In addition, neurotensin-immunoreactive fibers contact orexin neurons and could be implicated in maintaining their activity (Leinninger et al., 2011; Furutani et al., 2013).

Inhibitory inputs to orexin neurons also derive from multiple sources, including, within the LH, MCH neurons, as dealt with below. GABAergic afferents include somatostatin-containing varicosities, and these express the vesicular GABA transporter (VGAT) (Toossi et al., 2012). GABAergic neurons with electrophysiological properties distinct from those of orexin and MCH neurons have been reported in the LH (Karnani et al., 2013). These include neurons which express the leptin receptor (Leinninger et al., 2009) and directly innervate orexin neurons (Louis et al., 2010). Neurons which express the gut hormone ghrelin also suppress orexin neuron activity (Horvath et al., 2012). A recent report has confirmed that input to orexin neurons derives from a variety of GABAergic neurons within the LH (Ferrari et al., 2018). This local inhibitory input is depressed by acetylcholine; furthermore dynorphin, likely to be released locally from orexin neurons, inhibits orexin neurons directly but also disinhibits them indirectly by depressing the input derived from local GABAergic neurons (Ferrari et al., 2018).

In addition, inhibitory functional glycine receptors and glycinergic synapses onto orexin neurons have been identified by electrophysiology and immuno-electron microscopy (Hondo et al., 2011).

Monoamine neurotransmitters such as dopamine, noradrenaline, and serotonin also regulate the state-dependent activities of orexin neurons through inhibition via their receptors, such as D1/D2- (Bubser et al., 2005), alpha 2-adrenergic- (Li and van den Pol, 2005) and 5-HT1A- receptors (Muraki, 2004), respectively.

#### The Interplay With MCH Neurons

These cells are of special interest because they have been implicated in functions regulated also by orexin neurons, such as energy balance and sleep (Guyon et al., 2009; Bittencourt, 2011; Konadhode et al., 2013; Ferreira et al., 2017), autonomic functions (Adamantidis and de Lecea, 2009) and emotional behavior (Blouin et al., 2013).

Melanin-concentrating hormone neurons are distributed over other areas besides the LH (Bittencourt, 2011) but are largely intermingled with orexin neurons in the LH. These two cell groups play opposite roles in energy balance and especially in the regulation of sleep-wake states. MCH neurons promote sleep: they are active during rapid eye movement (REM) sleep, inactive during wakefulness (Hassani et al., 2009), and their optogenetic activation leads to REM and non-REM sleep in rats (Blanco-Centurion et al., 2016). Of note, at variance with orexin neurons MCH neurons are spared in the brain of narcoleptics (Thannickal et al., 2000, 2009).

Whole brain-mapping in mice has shown that afferents to MCH and orexin neuronal populations share many common sources, though it is not known whether the same cells innervate both peptidergic populations (González et al., 2016). MCH efferents are widely distributed and their axon terminals establish symmetric contacts (Bittencourt, 2011). In the local LH network they contribute to the synaptic inputs to orexin neurons (Bayer et al., 2002; Guan et al., 2002; Burt et al., 2011) exerting on them an inhibitory effect (Konadhode et al., 2015). Most MCH cells express GAD and are therefore GABAergic, but with the puzzling feature that they do not express VGAT (review in Herrera et al., 2017), as also confirmed by recent findings in transgenic mice (Blanco-Centurion et al., 2018) and by single-cell profiling indicating that GABA release from MCH neurons could either be non-synaptic or occur via non-canonical pathways (Mickelsen et al., 2017).

## Diurnal Changes of Orexin Neuron Activity

Given the role of the LH in physiological functions critical for survival which undergo substantial changes during 24 h (e.g., food intake and metabolism, vigilance states), it may not be surprising that diurnal (i.e., happening over a period of a day: day versus night), daily (i.e., happening every day) and circadian (*circa diem*: recurring over a 24 h cycle) changes have been reported in the LH. For example, gene profiling in mice from tissue punches collected from VPLO, suprachiasmatic nucleus (SCN) and LH has shown a diurnal fluctuation in gene expression, with difference and overlap in these regions between day and night (Gerstner et al., 2006).

It is worth recalling, in this respect, that functional, cellular, molecular fluctuations in the hypothalamus during 24 h have been related in different studies to day and night, to the animal’s rest and locomotor activity, and/or to vigilance states. In laboratory rodents, which are nocturnal, the light period corresponds to that of rest and predominant sleep, and the period of darkness to activity and wake predominance. The definition of vigilance states requires, however, electrophysiological characterization.

State-dependent behavior, in which all behavioral performances are inscribed, consists of three vigilance states: wake, REM sleep (also called paradoxical sleep, PS), non-REM sleep (also referred to in rodents as slow wave sleep, SWS), defined by neurophysiological parameters and, in particular electroencephalogram (EEG) recording. In model organisms such as the zebrafish, in which EEG cannot be recorded, sleep-like state is defined by behavioral criteria, based on the spontaneous occurrence of immobility with a circadian rhythm and under homeostatic control (Elbaz et al., 2013).

It should be noted, however, that in many studies of molecular, cellular or electrophysiological features of orexin neurons the time of the day of the study (including the time of the animals’ sacrifice, the time of preparation of brain slices, etc.) is not specified in the methodology, which therefore hampers a correlation with time of the day-dependent parameters.

### Neuronal Firing Profile

Most neuronal populations (such as dopaminergic, GABAergic, cholinergic, serotonergic neurons) in the sleep-wake network show diurnal variations in the profile of discharge that vary in strict dependency on the release of their neurotransmitter (Tyree and de Lecea, 2017). The interactions between different neuronal populations and their specific daily fluctuations result in variations of EEG pattern which characterize different behavioral stages (SWS, REM sleep, or wakefulness).

**FIGURE 1:**
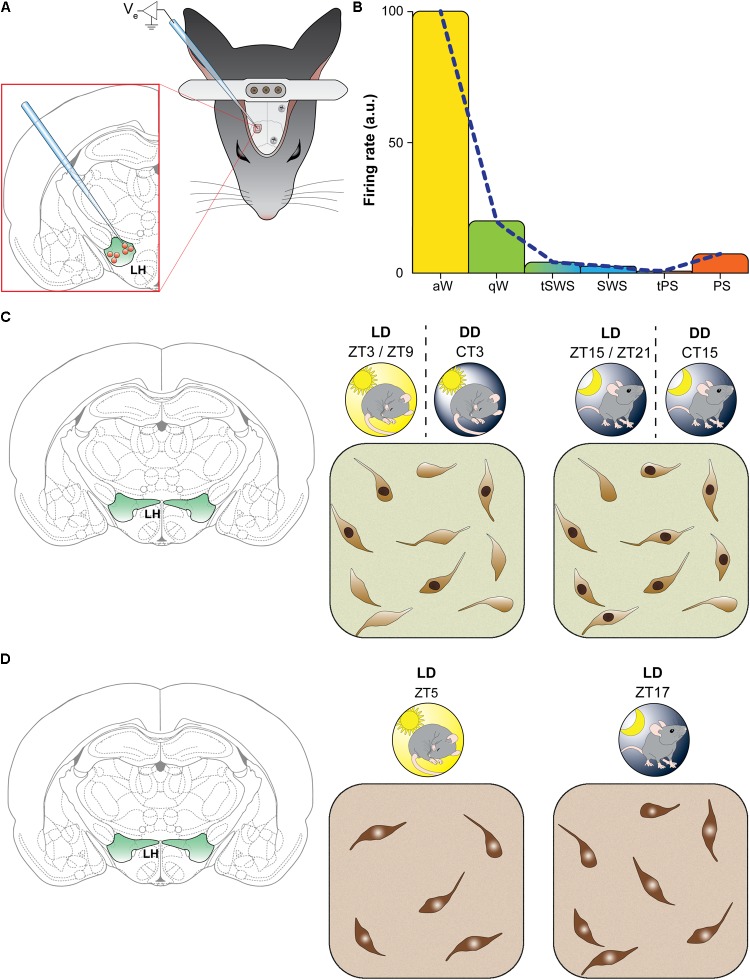
The activity of orexin neurons shows a diurnal fluctuation. **(A,B)** Neuronal firing rate. **(A)** Schematic representation of methodology used by Lee et al. (2005). Rats were implanted with epidural electroencephalographic electrodes (gray screws on skull) and with a metal bar for holding the head fixed while recording. A hole was drilled in skull to insert a glass micropipette for recording single unit from orexin neurons in the lateral hypothalamus. **(B)** Representative results found by Lee et al. (2005): the firing rate in arbitrary units (a.u.) of orexin-positive neurons varied significantly as a function of vigilance states. During active wakefulness (aW, in yellow) firing rate was highest and decreased during quiet wakefulness (qW, in green). The discharge was virtually absent during transitions between wakefulness and slow wave sleep (tSWS), during slow wave sleep (SWS, in blue) as well as during transitions between SWS and paradoxical sleep (tPS). During paradoxical sleep (PS, in orange) spike rate showed a little increase. Similar results was reported in rats by Mileykovskiy et al. (2005). **(C,D)** The figurines indicate laboratory rodents (findings depicted in C were obtained in rats, and those depicted in D in mice). LD indicates 12 h light/dark cycle entrainment, under which hours are referred to as Zeitgeber time (ZT; ZT0 corresponds to the lights-on time). DD indicates constant darkness, under which hours are referred to as circadian time (CT), corresponding to subjective light time (indicated by the sun in DD) and subjective dark time. **(C)** Spontaneous Fos induction. Schematic illustration of results found by Estabrooke et al. (2001). Double-label immunohistochemistry was performed to measure Fos expression in orexin neurons of the perifornical area. Under the LD cycle orexin-positive neurons showed higher (about two times) Fos immunoreactivity during the night (ZT15-ZT21; on the right) than during the day (ZT3-ZT9; on the left). The same pattern of activation still occurred in DD, indicating an endogenous rhythm. **(D)** Orexin synthesis: Schematic illustration of results found by McGregor et al. (2017). Immunohistochemistry was performed to count orexin neurons, and the number by orexin-positive neurons was found to be higher (about 24% increase) during the night (LD, ZT17, on the right) than the day (LD, ZT5, on the left).

Consistently with a pivotal role of orexin neurons for arousal promotion and maintenance, in rats entrained under the LD cycle, single unit recordings across the sleep-wake cycle have revealed a precise profile of discharge (Lee et al., 2005). Thus, orexin neurons show a relatively high discharge rate during active waking, the firing rate decreases ∼6 times during quiet wakefulness, and they become virtually silent during SWS and the transitions between quiet wake and SWS, as well as between SWS and REM sleep. During REM sleep, orexin neurons remain relatively silent, with an increase of firing rate only at the end of REM sleep episodes (Lee et al., 2005) (**Figures 1A,B**).

The same pattern of spiking was reported in rats (Mileykovskiy et al., 2005) and in mice (Takahashi et al., 2008) by means of extracellular unit recordings. In these studies, orexin neurons were found to be highly active during active wakefulness, decrease their firing activity during quiet wakefulness and become relatively silent during quiet SWS and tonic periods of REM sleep, with occasional discharges during phasic periods of REM sleep. Moreover, a correlation with electromyogram (EMG) activity, and therefore with muscle tone, was shown (Mileykovskiy et al., 2005).

### Spontaneous Fos Induction

Other sets of data derive from the investigation of Fos expression. This nuclear protein, encoded by the immediate early gene c-*fos*, is a widely used marker of neuronal activity, induced by different stimuli (Herdegen and Leah, 1998). However, Fos is also expressed in basal conditions in different cell groups of the brain, with a spontaneous oscillation during 24 h (Grassi-Zucconi et al., 1993, 1994; Cirelli et al., 1995).

In the LH, Fos is expressed in basal conditions in orexin, MCH, non-orexin and non-MCH neurons, as shown in rats (Verret et al., 2003; Modirrousta et al., 2005). In undisturbed rats maintained under the LD cycle, the number of orexin neurons showing Fos-immunoreactive nuclei is higher during night than day (Estabrooke et al., 2001) (**Figure 1C**). This was also documented when rats were maintained under constant darkness, indicating an endogenous oscillation (Estabrooke et al., 2001) (**Figure 1C**). In addition, EEG recording showed a positive correlation between Fos expression in orexin neurons and the amount of wakefulness and a negative correlation with the amount of sleep (non-REM and REM sleep) in the 2 h preceding the animal’s sacrifice (Estabrooke et al., 2001). During the light period, however, orexin neurons can be activated, as shown by Fos induction, when mice are working for reward (McGregor et al., 2011), and are activated during positively reinforced tasks in mice exposed to constant light (Marston et al., 2008), further supporting a motivational specificity of the orexin arousal function.

Fos expression in a higher number of orexin neurons during the period of activity than rest was also seen in diurnal rodents (Martínez et al., 2002; Kodama et al., 2005).

The use of Fos as a tool to disentangle the effects of wake and locomotor activity in rats suggested an association of locomotor activity, rather that waking *per* *se*, with increased orexin neurotransmission (España et al., 2003). This issue has been examined also with other approaches, as it will be discussed further.

### Orexin Synthesis and Release

It has been generally assumed that the number of orexin neurons detectable by immunocytochemistry does not vary across different behavioral states. A recent study has instead reported a diurnal variation in the number of immunolabeled orexin neurons (McGregor et al., 2017) (**Figure 1D**). A significantly greater number (24% increase) of orexin-immunopositive neurons, without significant changes in soma size, was found in undisturbed mice sacrificed during the dark phase compared to the light phase. The finding indicates that orexin expression does not reach the threshold for immunocytochemical detection in all orexin neurons during the phase of the animal’s rest. In contrast, there was no significant difference in the number of MCH-immunoreactive neurons between the animals in either phase, but a 15% increase in the soma size of MCH cells was found during the light phase compared to the dark phase (McGregor et al., 2017).

Other lines of evidence of diurnal fluctuation concern orexin synthesis. An initial study (Taheri et al., 2000) has reported in rats a diurnal variation in *prepro-orexin* mRNA in the hypothalamus and orexin immunoreactivity (measured by radioimmunoassay in brain homogenates) with a decrease during the day and an increase at night, in the preoptic/anterior hypothalamus and the pons, but not in other brain areas. Microdialysis and EEG (polysomnography) recordings showed a diurnal fluctuation of extracellular levels of orexin in the LH and medial thalamus of rats, with increase during the period of darkness and a decrease during the light period (Yoshida et al., 2001). In this study, in which the animals were also sleep-deprived for 6 h, orexin level was found to be dissociated from the amount of sleep and wake, favoring a role of orexin release in homeostatic processes. In cats in which vigilance states were characterized by EEG, local release of orexin measured with microdialysis was found to peak during active waking (Kiyashchenko et al., 2002).

Consistent with the role of orexin neurons in wakefulness and energy homeostasis, orexin level in the CSF of rats was found to be high during the period of darkness with a decrease in the light period (Fujiki et al., 2001; Desarnaud et al., 2004), and an increase after sleep deprivation (Desarnaud et al., 2004), as well as after food deprivation (Fujiki et al., 2001). In the cat, orexin level in the CSF was markedly higher during active waking than quiet waking, favoring a correlation with motor activity (Kiyashchenko et al., 2002).

In squirrel monkey, a diurnal non-human primate, the orexin level of cisternal CSF was found to exhibit a strong diurnal pattern with increasing concentration during the wake period and maximum levels in the early evening (the latter third of the wake period), falling then throughout the night (Zeitzer et al., 2003). Prolongation of daytime wakefulness for about 4 h did not result in significant change in CSF orexin levels, whereas sleep deprivation during the night resulted in significant increase in CSF orexin levels. Orexin thus appeared to act as a reactive homeostatic signal, with a circadian contribution opposite to sleep drive (Zeitzer et al., 2003).

In humans, the interpretation of findings on orexin levels in the CSF, which seem to increase during sleep, is complicated by the sampling times and by the methodology, represented by the lumbar puncture (in contrast to sampling from the cisterna magna in experimental animals), due to which the temporal profile may differ, being delayed, from that of brain regions (Salomon et al., 2003; Grady et al., 2006). It is interesting to note, however, that seasonal changes in orexin level in the CSF have been reported in humans, with 10–12% change from winter to summer, correlating with day length period but not with measures of sleep length (Boddum et al., 2016).

Diurnal variations of postsynaptic effects of orexin, especially relevant in the present context, have been studied in hippocampal subfields (Perin et al., 2014). In acute hippocampal slices prepared from rats during daytime (the period of rest), exogenous orexin was found to exert an inhibitory effect on mossy fibers synapses. This inhibition, mediated by OX2R, was absent in slices prepared at the end of the night (Perin et al., 2014). The findings point to time-of-day-dependent postsynaptic effects of orexin release, at least in some of its targets. In the SCN, orexin release exerts an inhibitory effect during both day and night, but with different postsynaptic mechanisms (Belle et al., 2014).

### Plasticity in the Wiring of Orexin Neurons After Food Intake and Sleep/Wake Manipulations

Experimental manipulations of the main functions in which orexin is involved, namely food intake and wake regulation, have pointed out a remarkable synaptic plasticity.

The first data indicating that changes in the nutritional status are able to affect synaptic plasticity of orexin neurons and their connectivity derived from synaptology and electrophysiological studies during fasting and re-feeding in mice (Horvath and Gao, 2005). Fasting was found to increase the number of excitatory inputs to orexin somata with recruitment of additional terminals, reaching a 10:1 ratio of excitatory versus inhibitory terminals on orexin cell bodies; re-feeding after fasting partially reversed these fasting-induced changes. Since administration of leptin prevented fasting-induced plasticity, it was proposed that the rewiring of orexin neurons could result from multiple humoral and neuronal signals, and that synaptic changes in the orexin circuitry could underlie functional alterations such as insomnia, and metabolic disturbances including obesity (Horvath and Gao, 2005).

Indeed, an excitatory-to-inhibitory switch of synapses on orexin somata has been reported in obese mice, both in the genetic model of obesity represented by leptin-deficient *ob/ob* mice and in mice subjected to high-fat diet (HFD), a model of diet-induced obesity (Cristino et al., 2013). In this study, in which parameters related to retrograde endocannabinoid signaling were examined, presynaptic inputs to orexin cell bodies were characterized by double immunofluorescence of synaptophysin combined with VGAT or VGluT2 and by the ultrastructural investigation of symmetric and asymmetric synaptic contacts. In obese mice, the perisomatic innervation was found to change from predominantly excitatory to predominantly inhibitory with consequent endocannabinoid-mediated disinhibition of orexin neurons and elevation of orexin trafficking and release to many target areas.

The mechanism of such synaptic remodeling was also investigated (Cristino et al., 2013), highlighting in obese mice a role of lack of leptin in the arcuate nucleus, the source of most of the fibers which express the (endo)cannabinoid receptor type 1 (CB1) and innervate orexin neurons. Treatment with leptin reversed the synaptic remodeling but only in *ob/ob* mice and not in HFD mice, indicating that this phenomenon is a consequence of leptin deficiency or leptin resistance in the arcuate nucleus. Interestingly, possibly because *ob/ob* mice lack endogenous leptin when weaned but receive leptin from their heterozygous mothers during lactation, synapse remodeling at orexin neurons only occurred after weaning and was reversed by exogenous leptin injection (Cristino et al., 2013; Becker et al., 2017).

Consistent with these findings and with data indicating that experimental exposure to HFD induces various forms of functional and structural plasticity within the reward circuitry (Sharma et al., 2013), a recent report in rats has pointed out plastic changes in orexin neurons following exposure to HFD (a palatable high fat Western diet) (Linehan et al., 2018). Short-term exposure to HFD increased the level of glutamate, thereby priming excitatory synapses onto orexin neurons to undergo activity-dependent long-term depression. However, this priming was transient as it disappeared following prolonged exposure to HFD. The observed changes may thus alter orexin network properties and could also underlie their physiological role in reward-based feeding (Linehan et al., 2018).

Dynamic remodeling of orexin neuron wiring has also been described in response to changing physiological needs such as sleep. Electrophysiological analysis in mice showed an increased strength of glutamatergic synapses on orexin neurons following prolonged wakefulness induced by modafinil treatment or 4 h sleep deprivation, and ultrastructural analysis revealed a significant increase in the number of asymmetric synapses in modafinil-treated mice (Rao et al., 2007). Accordingly, sleep-deprived or modafinil-treated mice showed increased locomotor activity. The findings confirmed that physiological or environmental cues lead to a reorganization of excitatory synapses on orexin neurons and, in turn, experience-dependent plasticity in orexin neurons may underlie prolonged wakefulness (Rao et al., 2007, 2008).

### Diurnal Changes in the Wiring of Orexin Cell Bodies in Basal Conditions

The issue of the synaptology of orexin neurons in basal conditions has been examined in the model organism zebrafish, a diurnal vertebrate, and in mice.

About 16 orexin neurons are present in zebrafish larvae and about 60 in the adult animals. The larvae are transparent, thus allowing *in situ* live imaging studies. Orexin-A in zebrafish shares 32% amino acid sequence with that of the human peptide, and the only identified OXR corresponds structurally to the mammalian OX2R (Elbaz et al., 2013). Orexin neurons are glutamatergic also in zebrafish (Appelbaum et al., 2009), and their expression during arousal and their circuitry point to a role of orexin in vigilance state transitions also in this species (Elbaz et al., 2013).

*In vivo* two-photon imaging revealed that the number of presynaptic terminals of orexin neurons projecting to the pineal gland show a circadian rhythmicity in both LD and constant darkness conditions, excluding a light exposure effect. The number of terminals was found to be highest over the subjective light phase (activity period) and to decrease during the subjective dark phase (rest period), highlighting a circadian control of the orexin synapse arrangement (Appelbaum et al., 2010). Moreover, 6 h sleep deprivation in zebrafish resulted in an increase of ∼17% of orexin terminals, which returned to control levels in the sleep recovery period (Appelbaum et al., 2010).

Concerning orexin neuron wiring, the number of inhibitory synapses on orexin dendrites was found to peak in zebrafish larvae during the night, i.e., during the period of rest of this animal, and there were no changes under constant light or dark conditions (Elbaz et al., 2017). During night, sleep deprivation reduced the synapse number, which increased during the post-deprivation daytime sleep (Elbaz et al., 2017). This rhythmic plasticity of inhibitory synaptic contacts on orexin neuron dendrites was found to be modulated by consolidated wake and sleep, and therefore independent from the circadian clock (Elbaz et al., 2017).

**FIGURE 2:**
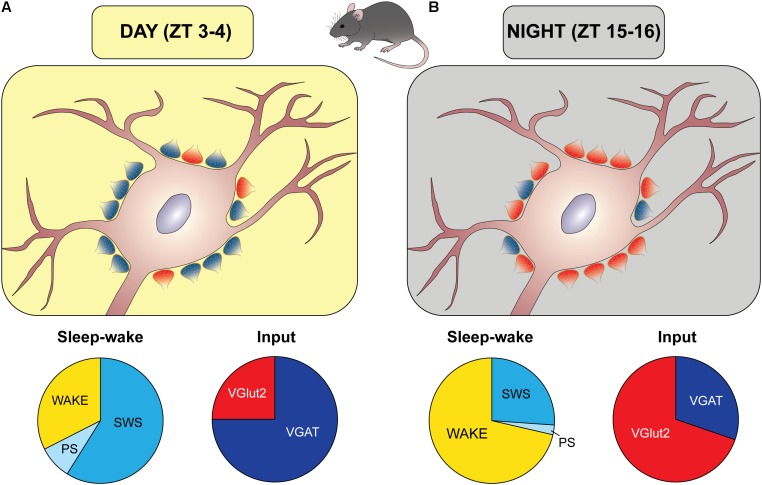
Diurnal remodeling of the excitatory/inhibitory input to orexin cell bodies. Schematic representation of synaptic organization (excitatory/inhibitory balance) during the light period **(A)** and the period of darkness **(B)**, based on the results by Laperchia et al. (2017) in mice. *Zeitgeber* time (ZT) 0 corresponds to the lights-on time. During the day **(A)** there was a prevalence of inhibitory synapses (blue boutons), as determined by immunopositivity to the presynaptic vesicular GABA transporter (VGAT) and postsynaptic gephyrin, when matched mice spent most of this time asleep (slow wave sleep, SWS, and paradoxical sleep, PS), as depicted by the apple-pie chart. An opposite arrangement was found during the night, when excitatory synapses (red boutons), as determined by immunopositivity to the presynaptic vesicular glutamate transporter (VGluT)2 and postsynaptic density protein 95 (PSD95), prevailed, and matched mice spent most of this period awake.

The issue of the diurnal balance between excitatory and inhibitory inputs onto orexin somata in undisturbed mice has been recently examined in our laboratories (Laperchia et al., 2017). In this study, the animals were sampled at two time points in antiphase during day and night. To avoid any potential stressful condition, EEG was recorded in matched mice showing, in the 2 h preceding sacrifice, a predominance of sleep (about 70% of this time interval) during day and a predominance of wake (about 71%) during night (**Figure 2**). To characterize excitatory and inhibitory terminals, the input to orexin cell bodies was visualized using the presynaptic marker synaptophysin combined with VGAT or VGluT2, as well as with each vesicular neurotransmitter transporter combined with the respective postsynaptic scaffold protein (VGAT with gephyrin for inhibitory synapses, VGluT2 with PSD95 for excitatory synapses). A day/night reorganization of the balance between excitatory and inhibitory inputs to orexin cell bodies was observed, without a variation of their total number, with a predominance of inhibitory synapses during the day (about 75%) and a predominance of excitatory synapses during the night (about 70%) (**Figure 2**).

This set of data requires implementation. From the behavioral point of view, although the sleep and wake percentages in the hours preceding sacrifice showed very little interindividual variability in matched animals, vigilance state needs to be characterized in the same animals, and videorecording experiments are currently ongoing to this purpose. Furthermore, experiments under constant darkness need to be performed to verify an endogenous oscillation.

At the subcellular level, excitatory and inhibitory inputs onto orexin neuron dendrites in the same paradigm remain to be examined. It should, however, be considered that the neuronal soma is the site of integration of all inputs and is a critical domain given the proximity to the site of action potential generation (Spruston, 2008).

At the synaptic level, electron microscopy is the gold standard for the visualization of synapses. Furthermore, although the contact with the postsynaptic scaffolding protein indicates a functional synapse (Wouterlood et al., 2003, 2007; Henny and Jones, 2006; Toossi et al., 2012), morphological approaches cannot definitely ascertain whether synaptic contacts are functional. It should also be considered that VGluT2 and VGAT do not visualize terminals releasing other neurotransmitters, such as cholinergic ones. Concerning GABAergic input, VGAT is the transporter for both GABA and glycine (Wojcik et al., 2006), but, as mentioned previously, VGAT is not expressed by all GABAergic terminals which contact orexin neurons, and, in particular, is not expressed by MCH terminals.

Even considering these limitations, the above findings in mice show a time-of-day-dependent fluctuation in the perisomatic innervation of orexin neurons, and indicate a relationship of this process with sleep and wake predominance.

## Which Mechanism/S for Diurnal Regulation of Orexin Neurons?

### Circadian and Homeostatic Regulation

Physiological functions are regulated by the strict interplay between the need of maintain an internal milieu (Bernard, 1865) and the need to anticipate the fluctuations of the external environment. The two main actors of this balance are the homeostatic process, by which physiological variables are regulated to remain near a defined value over time (Cannon, 1929), and circadian regulation (Halberg et al., 1959) that favors changes for an optimal functioning at the proper time of the day. The interaction between circadian and homeostatic components determines the timing of sleep and wake according to the influential “two-process model” (Borbély, 1982; Borbély et al., 2016). In both humans and animals, a prolonged period of wakefulness exerts a greater sleep pressure and longer and deeper episodes of the subsequent sleep (homeostatic component, process S). On the other side, the circadian component (process C) is independent from sleep and wake duration and defines the daily fluctuation of sleep propensity.

Evidence points to the daily fluctuation of orexin level in the CSF as an endogenous rhythm: this fluctuation was robustly maintained in rats entrained under dim red light (Deboer et al., 2004) as well as under constant light or constant darkness (Zhang et al., 2004). As the other endogenous rhythms, this orexin fluctuation in mammals is under the control of the master circadian pacemaker, the SCN. Lesions of the SCN abolish the daily fluctuation of CSF orexin level in rats under different light exposure conditions (Deboer et al., 2004; Zhang et al., 2004).

Concerning the complex issue of the relationships between orexin neurons and the biological clock, anterograde tracing data have suggested a polysynaptic link of the SCN with orexin neurons (Deurveilher and Semba, 2005), consistently with other polysynaptic SCN efferent pathways (Saper et al., 2005). As for the reciprocal connectivity, initial mapping showed a dense orexin-immunoreactive terminal field around, but not within, the SCN (Peyron et al., 1998) but direct orexin innervation of the SCN has then been shown (Belle et al., 2014). As also mentioned previously, orexin exerts a suppression on SCN activity, which shows a diurnal fluctuation, with predominantly GABA-mediated presynaptic actions during the day which switch to direct postsynaptic effects during the night (Belle et al., 2014).

Interestingly, findings on orexin level in the CSF of rats after different manipulations (Yoshida et al., 2001; Deboer et al., 2004) have indicated that the SCN is not the only component that regulates orexin fluctuation, which should therefore involve also a homeostatic process.

In zebrafish, the above-mentioned data indicate that orexin activity, release and synaptic re-arrangement are controlled by both circadian and homeostatic components. Homeostatic mechanisms have also been implicated in Fos expression in orexin neurons after sleep deprivation and sleep recovery in rats (Modirrousta et al., 2005), and in the expression of the subunit α1 of GABA_A_ receptor (GABA_A_R) in orexin cell bodies after sleep deprivation in mice, confirmed by *in vitro* electrophysiology (Matsuki et al., 2015). Similarly, the expression and density of both GABA_A_R and GABA_B_R on orexin neurons strongly increase in mice after sleep deprivation and return to baseline during sleep recovery (Toossi et al., 2016). Increase in the activity of orexin neurons during the dark period in mice (Modirrousta et al., 2005) results in up-regulation of GABARs on orexin neurons (Toossi et al., 2016), facilitating their inhibition according to the homeostatic regulation of synaptic plasticity (Turrigiano, 1999). It thus seems that the interplay between circadian and homeostatic processes plays a pivotal role in the daily fluctuation of the orexin system.

As also mentioned previously, the contribution of wakefulness *per se* versus locomotor activity has been discussed in several paradigms. Some lines of evidence point indeed to a correlation of orexin activity with locomotor activity. Pivotal experiments showed that intracerebroventricular injection of exogenous orexin in rats increases locomotor activity (Hagan et al., 1999; Ida et al., 1999; Piper et al., 2000), but did not disentangle whether this is a direct effect of locomotion or a secondary effect due to increased wakefulness. The amount of locomotor activity rather than wakefulness has been related to orexin levels in different sets of experiments in rats and dogs (Wu et al., 2002; Martins et al., 2004; Zhang et al., 2004). However, experiments in squirrel monkeys have indicated that locomotion is not necessary for the daily fluctuation of CSF orexin levels (Zeitzer et al., 2004).

It should also be considered that it is very demanding to clearly dissociate the influences of locomotor activity and wakefulness, since motor activity occurs especially during wakefulness. Overall, however, this distinction may not be necessary since, on the basis of their links with sleep-wake-regulatory as well as premotor and motor centers, orexin neurons may control both wakefulness and locomotor activity in a coordinated manner.

### Multiple Players in the “Chronoconnectivity” of Orexin Neurons?

Synaptic mechanisms for a time-of-day dependent and sleep-wake-related daily remodeling could involve different signaling pathways and multiple elements.

Among chemical regulators potentially involved in the functional “chronoconnectivity” of orexin neurons, endocannabinoids and hormones are likely to play a role. Endocannabinoids are emerging as master players of fast (i.e., non-genomic) and energy-related fine-tuning of synaptic inputs to orexin neurons, depending on CB1 expression at glutamatergic or GABAergic terminals. In this scenario, fasting-related reduction of leptin levels seems to control arousal by increasing the activity of orexin neurons, which are innervated by CB1-expressing excitatory inputs, and are a main source of the endocannabinoid 2-arachidonoylglycerol (Huang et al., 2007). Supporting endocannabinoid-regulated activity of orexin release, a positive correlation has been described in healthy humans between the circadian rhythm of 2-arachidonoylglycerol serum level and sleep timing or caloric intake (Hanlon et al., 2015, 2016; Cedernaes et al., 2016). Circulating hormones such as leptin and ghrelin, which regulate metabolism and, as mentioned previously, influence orexin neuron activity also through neural pathways, are under circadian regulation (Challet, 2015). Circadian rhythms of synaptic morphology can be regulated by hormonal release (Frank, 2016).

“Chronoconnectivity” of orexin neuron wiring could potentially involve a timing-related regulation of neurons capable of glutamate and GABA co-release. This has not been examined in sources of orexin neuron innervation, but co-release of these fast neurotransmitters has been reported at other sites (Beltrán and Gutiérrez, 2012; Shabel et al., 2014; Ntamati and Lüscher, 2016; Galván and Gutiérrez, 2017).

Coexistence of VGAT and VGluT1 or VGluT2 has been reported in the hypothalamic periventricular nucleus (Ottem, 2004), hippocampal dentate gyrus (Boulland et al., 2009) and neocortex (Fattorini et al., 2009), and it has been shown in immunoisolated synaptic vesicles that VGAT can transport glutamate besides GABA (Zander et al., 2010). However, synaptophysin and VGAT expression in synaptic vesicles in the brain does not undergo a circadian oscillation, while VGluT2 seems to be rearranged at the synaptic level during 24 h, but without variation of the overall expression in presynaptic terminals (Darna et al., 2009). It also remains that the inhibitory or excitatory synaptic phenotype is determined by postsynaptic scaffolding proteins besides the marker/s of presynaptic release. In this context, activity-dependent plasticity of gephyrin at GABAergic synapses has been reported (Flores et al., 2015). Furthermore, neuronal plasticity can be regulated by circadian rhythms of cytoskeletal components (e.g., Petsakou et al., 2015).

Considering synaptic components besides the pre- and post-synaptic elements, it is now known that perisynaptic astrocytic sheaths are important local players, which has led to the concept of a tripartite synapse (Araque et al., 1999) and that extracellular matrix (ECM) structures interact with neuronal and glial synaptic components, which has led to the concept of a quadripartite synapse (Dityatev et al., 2010).

Astrocytes take part in the local network of regulation of orexin neuron activity within the LH (Burt et al., 2011). Knock-out of connexin-43 gap junction subunits in astrocytes throughout the brain led to the silencing of orexin neurons, thereby causing excessive sleepiness and fragmented wakefulness (Clasadonte et al., 2017). Modulation and negative feedback regulation of excitatory glutamatergic inputs and the release of astrocyte-derived factors, such as lactate and adenosine triphosphate (ATP), can affect the excitability of orexin neurons. It has been shown that ATP release from cultured astrocytes is under circadian control (Marpegan et al., 2011). Orexin neurons utilize lactate derived from astrocytes as energy substrate (Parsons and Hirasawa, 2011), and glutamatergic transmission has been proposed to stimulate orexin neurons via the astrocyte-neuron lactate shuttle mediated through monocarboxylate transporters (Burt et al., 2011). This role of astrocytes as energy substrate is critical for the activity of orexin neurons. Perisomatic appositions expressing glutamate transporter 1, the main astrocytic transporter which plays a key role in glutamatergic transmission, decrease around orexin neurons and increase around MCH neurons after sleep deprivation in the rat (Briggs et al., 2018).

In their surveillance function, microglial cells continuously extend and retract their branches, contacting also synapses (Miyamoto et al., 2013). Diurnal changes of the overall extent of microglial branches have been observed in the cerebral cortex of mice in basal conditions (Hayashi et al., 2013b). Investigations on the molecular mechanisms underlying the diurnal morphological changes of cortical microglia have pointed out a regulation by clock genes (Hayashi et al., 2013a,b) and therefore an “intrinsic microglial molecular clock” (Hayashi et al., 2013a). This was found to drive the circadian expression of cathepsin S, a microglia-specific protease which degrades ECM molecules (Hayashi et al., 2013a), thus implicating ECM components in the extension and retraction of microglial cell processes at different times of the day.

On the other hand, ECM components are main players in synaptic plasticity phenomena, not only during development but also in the adult brain (Dityatev et al., 2010; Song and Dityatev, 2017; Ferrer-Ferrer and Dityatev, 2018). The organization of the ECM has been investigated in the anterior hypothalamus (Horii-Hayashi et al., 2017) but remains to be investigated in the LH.

Overall, diurnal changes of glial cells and ECM in the LH in basal conditions await for further studies, which could reveal in this region an orchestra of multiple elements engaged in synaptic remodeling.

## Concluding Remarks

The data here reviewed point to a daily fluctuation of excitatory and inhibitory inputs to orexin neurons as a feature of the logic of this vast network to face dynamic physiological demands. This envisages a “chronoconnectivity” which may involve different sets of inputs and may underlie diurnal fluctuation of neuronal firing and overall neuron activity.

Such conceptual framework is different from the synaptic homeostasis processes hypothesized during sleep and wake (Tononi and Cirelli, 2003, 2014). The synaptic homeostasis hypothesis is centered on the variation of synaptic strength leading to synaptic potentiation during wake and synaptic depression during sleep (Tononi and Cirelli, 2003, 2014), but does not involve neuronal firing rates (Cirelli, 2017). Synaptic homeostatic changes during sleep and wake could be subserved by circadian synaptic changes, as observed in synaptic morphology in the somatosensory cortex (Jasinska et al., 2015), and in the size of cortical dendritic spines and area of axon-spine interface during sleep and wake (De Vivo et al., 2017). “Chronoconnectivity” implies instead time of day-dependent and sleep-wake-dependent fluctuation of firing rate and connectivity. In the wiring of orexin neurons, this could involve extra- and intra-hypothalamic mechanisms which remain to be elucidated.

Of note, changes of neuronal firing in different phases of the sleep-wake cycle is a property of many cell groups in the distributed network of sleep-wake regulation (Scammell et al., 2017), as also highlighted by optogenetic studies (Tyree and de Lecea, 2017). The question thus arises: could neuron wiring fluctuation represent a general property of this network?

## Author Contributions

IA wrote the first draft of the manuscript. FDG and LC contributed to various parts of the text and FDG prepared the figures. MB contributed to the theoretical framework and prepared the final manuscript version.

## Conflict of Interest Statement

The authors declare that the research was conducted in the absence of any commercial or financial relationships that could be construed as a potential conflict of interest.
